# Faradaic Impedimetric Immunosensor for Label-Free Point-of-Care Detection of COVID-19 Antibodies Using Gold-Interdigitated Electrode Array

**DOI:** 10.3390/bios14010006

**Published:** 2023-12-22

**Authors:** Lian C. T. Shoute, Carmen L. Charlton, Jamil N. Kanji, Shawn Babiuk, Lorne Babiuk, Jie Chen

**Affiliations:** 1 Department of Electrical and Computer Engineering, University of Alberta, Edmonton, AB T6G 1H9, Canada; shoute1@ualberta.ca; 2 Department of Laboratory Medicine and Pathology, University of Alberta, Edmonton, AB T6G 1C9, Canada; carmen.charlton@albertaprecisionlabs.ca (C.L.C.); kanji1@ualberta.ca (J.N.K.); 3 Public Health Laboratory, Alberta Precision Laboratories, Calgary, AB T2N 1M7, Canada; 4 Li Ka Shing Institute for Virology, University of Alberta, Edmonton, AB T6G 2E1, Canada; 5 Division of Infectious Diseases, Department of Medicine, Cumming School of Medicine, University of Calgary, Calgary, AB T2N 4N1, Canada; 6 Department of Pathology & Laboratory Medicine, Cumming School of Medicine, University of Calgary, Calgary, AB T2N 4N1, Canada; 7 National Centre for Foreign Animal Disease, Canadian Food Inspection Agency, Winnipeg, MB R3E 3M4, Canada; shawn.babiuk@inspection.gc.ca; 8 Department of Immunology, University of Manitoba, Winnipeg, MB R3E 0T5, Canada; 9 Vaccine and Infectious Disease Organization, University of Alberta, Edmonton, AB T6G 2G3, Canada; lbabiuk@ualberta.ca; 10 Department of Biomedical Engineering, University of Alberta, Edmonton, AB T6G 1H9, Canada

**Keywords:** Faradaic detection, pinholes, baseline drift, label-free, interdigitated electrode microelectrode array, COVID-19 antibody detection

## Abstract

Label-free electrochemical biosensors have many desirable characteristics in terms of miniaturization, scalability, digitization, and other attributes associated with point-of-care (POC) applications. In the era of COVID-19 and pandemic preparedness, further development of such biosensors will be immensely beneficial for rapid testing and disease management. Label-free electrochemical biosensors often employ [Fe(CN)_6_]^−3/4^ redox probes to detect low-concentration target analytes as they dramatically enhance sensitivity. However, such Faradaic-based sensors are reported to experience baseline signal drift, which compromises the performance of these devices. Here, we describe the use of a mecaptohexanoic (MHA) self-assembled monolayer (SAM) modified Au-interdigitated electrode arrays (IDA) to investigate the origin of the baseline signal drift, developed a protocol to resolve the issue, and presented insights into the underlying mechanism on the working of label-free electrochemical biosensors. Using this protocol, we demonstrate the application of MHA SAM-modified Au-IDA for POC analysis of human serum samples. We describe the use of a label-free electrochemical biosensor based on covalently conjugated SARS-CoV-2 spike protein for POC detection of COVID-19 antibodies. The test requires a short incubation time (10 min), and has a sensitivity of 35.4/decade (35.4%/10 ng mL^−1^) and LOD of 21 ng/mL. Negligible cross reactivity to seasonal human coronavirus or other endogenous antibodies was observed. Our studies also show that Faradaic biosensors are ~17 times more sensitive than non-Faradaic biosensors. We believe the work presented here contributes to the fundamental understanding of the underlying mechanisms of baseline signal drift and will be applicable to future development of electrochemical biosensors for POC applications.

## 1. Introduction

Coronavirus disease 2019 (COVID-19) caused by severe acute respiratory syndrome coronavirus 2 (SARS-CoV-2) was first reported in late 2019 and declared as a pandemic on 11 March 2020 [1]. SARS-CoV-2 is closely related to the original SARS-CoV virus which is believed to be of zoonotic origin. Coronaviruses represent a large family of viruses common in human and many different species of animals and SARS-CoV-2 is the seventh known coronavirus to infect humans [2]. The COVID-19 pandemic is the most devastating global health crisis since the 1918 influenza pandemic and consequently resulted in a far-reaching economic crisis including the second largest global recession in recent history. The World Health Organization (WHO) current advisory is to maintain core SARS-CoV-2 surveillance activities [3,4].

A key step to pandemic management is preventing disease transmission through rapid testing and identification of infected individuals. POC diagnostic tools have proven to be highly valuable for global screening since no prior training is required. Population-wide testing is also recommended by the WHO and other experts who have called for increased testing as the best approach to monitor and slow the spread of SARS-CoV-2 [5,6,7,8]. The target analytes suitable for determining SARS-CoV-2 infection are viral RNA [9,10,11,12], viral antigen [12,13,14,15,16], and antibodies produced as part of the adaptive humoral response to viral infection [17,18,19,20,21,22,23]. The gold standard for diagnosing initial infection is real-time reverse transcriptase polymerase chain reaction (RT-PCR) which detects viral RNA or its genome [9,10,11,12]. Initial or active infection can also be diagnosed with antigen tests which detect viral structural proteins such as the nucleocapsid and the spike proteins of SARS-CoV-2 [13,14,15,16]. Antibody serology tests detect the presence of antibody that binds to a specific SARS-CoV-2 protein on the virus [17,18,19,20,21,22,23]. Since antibodies are produced by the immune system in response to viral infection, they can be detected only after seroconversion, and hence a positive serological test indicates both current and past infection. More importantly, the usefulness of antibody serology tests lies in their applications to the development and evaluation of therapeutic antibodies, vaccines, and identifying potential convalescent plasma donors in addition to epidemiological assessment and in the diagnosis of suspected cases which showed negative viral RNA or antigen tests. Further, serology tests have several advantages over nucleic acid and antigen tests including a much longer detection window, stability of antibodies, and the target analyte in this case is the antibody rather than active SARS-CoV-2. The standard laboratory serological diagnostic techniques include, enzyme-linked immunosorbent assays (ELISAs), immunofluorescence assays (IFAs), and chemiluminescence assays (CLIAs) [17,18,19,20,21,22,23].

At present, lateral flow immunoassay (LFIA) has become the method of choice for POC applications since the test is low-cost, simple, can be completed in a single step, and yields results in less than 15 min [24,25,26,27]. LFIA is an immunochromatographic device composed of four main sections: a cellulose sample pad, a glass fiber conjugate pad, a nitrocellulose detection pad with a printed test line and control line, and cellulose absorption pad to wick the sample. The conjugation pad stores gold nanoparticle–receptor conjugates which bind specifically to target analytes in the sample as it flows from the sample pad by capillary force. The test line on the detector pad has immobilized capture receptors which bind specifically to a different receptor on the target analyte, and the control line has immobilized secondary antibodies which bind specifically to the nanoparticle–receptor conjugates. The test result is a colorimetric read-out displayed as a change in color appearing at the test line which can be detected by the naked eye. However, the simplicity of LFIA is accompanied by several technical limitations. Notably, LFIA tests are orders of magnitude less sensitive and specific compared to standard laboratory techniques. LFIAs also lack digital connectivity for data collection and linkage to the health care system, thereby limiting their ability for monitoring the spread of disease.

Electrochemical biosensors are ideal for digital connectivity and miniaturization as the interaction of the analytes with the biorecognition element on the transducer is detected as an electrical signal unlike other biosensors which require a separate detection unit [28,29,30,31,32,33,34]. Further, they are compatible with label-free protocols and reported performances for the detection of many biomarkers are significantly better than that of gold standard ELISA methods. Electrochemical detection methods can be categorized into the following three major categories based on the measured property: amperometry/voltammetry, potentiometry, and impendence spectroscopy. Recently, microelectrodes based on IDAs are increasingly being used as transducers in electrochemical biosensors [35,36,37,38,39]. Microelectrodes consist of two addressable interdigitated comb-like electrodes and have many distinct advantages including fast response time, low capacitive current, high collection efficiency, and a higher signal-to-noise ratio. In addition, IDA in conjunction with impedimetric detection provides additional advantages in its ability to perform sensing without the need for a reference electrode which in turn facilitates the device miniaturization. In fact, reference electrodes are not essential for applying a stable potential to the working electrode when the measured currents are small (~µA) [40,41,42]. An electrochemical biosensor can be classified as either Faradaic or non-Faradaic, depending on the presence or absence of redox reagents in the solution for the detection of target analytes.

Recently, we have reported label-free non-Faradaic electrochemical impedance spectroscopy (EIS) detection of COVID-19 antibodies with the spike protein as the biorecognition element covalently attached to gold IDA transducer [43]. We demonstrated a limit-of detection (LOD) of 0.4 binding antibody units (BAU)/mL, where BAU is binding antibody units, which is similar to commercially available standard immunoassays for anti-SARS-CoV-2 immunoglobulins. Since Faradaic detection is known to provide a significantly enhanced signal amplification compared to non-Faradaic detection, the vast majority of electrochemical biosensors currently under development are based on Faradaic detection [44,45,46,47,48,49,50,51,52,53,54]. However, biosensors based on Faradaic detection using a [Fe(CN)_6_]^−3/4^ redox system are reported to have limitations arising from baseline signal drifts which are often not adequately addressed and can therefore compromise the reliability of the devices. Plausible origin of baseline signal drift for gold-electrode-based electrochemical biosensors has been attributed to etching of the gold by cyanide and chloride ions from [Fe(CN)_6_]^−3/4^ in phosphate-buffered saline [55,56,57,58,59], oxidation of thiolate head groups [60,61,62], spontaneous dissociation of Au-S bonds [63,64,65,66], Oswald ripening of the thiolate SAM [67,68], and reorganization of defects (pinholes and collapsed sites) in the SAM [62,64,69,70]. Thus, a better fundamental understanding of the origin of the baseline signal drift is required in order to address this challenge.

Recently, Kanyong and Davis [71] reported a two-step protocol that involves applying a cycling voltage followed by a subsequent solution-phase incubation. This protocol effectively mitigates the baseline signal drift observed in 11-mercaptoundecanoic acid (MUA)-functionalized gold electrodes. The imposed cycling voltage induces a torque on the thiolate head group driving reorganization of defects leading to increase in the number of pinholes with the concurrent decrease in the number of collapsed sites. The fractional coverage of the MUA SAM on the gold remains relatively unchanged based on the observed values of 0.999 and 0.994 for freshly prepared MUA SAMs and two-step treated SAMs, respectively. The incubation step facilitates the formation of a crystalline and homogeneous SAM and provides the biosensor with a highly suppressed baseline signal drift. In this paper, we report on the development of a simple approach which consists of incubating a MHA SAM-coated IDA in 5 mM [Fe(CN)_6_]^−3/4^ 1x Tris-buffered solution and applying bias voltage intermittently by scanning the EIS of the system. The voltage imposition on the sensor was achieved by recording the EIS spectrum using an excitation AC sinusoidal voltage of ±10 mV superimposed on a relatively high 0.4 V DC and scanning the frequency from 1 Hz to 0.1 MHz to facilitate the voltage induced SAM reorganization and suppress the baseline signal drifts. In this study we observed an initial rapid decrease in charge-transfer resistance (R_ct_) followed by relaxation to a relatively stable region where the baseline signal drift is highly suppressed and in this region the coefficient of variation of the R_ct_ was <3%. However, prolonged incubation in 5 mM [Fe(CN)_6_]^−3/4^ solution led to etching and dissolution of the gold fingers of the IDA. The observed experimental data showed a decrease in both the pinhole radius and their separation in the SAM coated IDA during the stabilization period thereby providing insights into the origin of the baseline signal drift. These results point to the operation of two concurrent mechanisms contributing to baseline drifts in MHA-coated IDA where the faster kinetics contribute to reorganization process dominating over the slower etching of the gold by CN^−^ ions, thereby providing a window of relative stability in the intermediate time scale. The MHA-functionalized gold IDAs with highly suppressed baseline signal drift were used to develop a label-free immuno-impedimetric biosensor with Faradaic current as the signal for detection and quantification of COVID-19 antibodies in human serum samples.

## 2. Materials and Methods

### 2.1. Materials and Reagents

Borofloat glass wafers were purchased from Swift Glass Co., Inc. Elmira Heights, NY. USA. 1-Mercapto-6-hexanoic acid 97% (MHA), N-(3-(dimethylamino)propyl)-N′-ethylcarbodiimide hydrochloride (EDC), N-hydroxysuccinimide 98% (NHS), 2-morpholinoethanesulfonic acid monohydrate(MES), ethanol 100% (EtOH), acetone (AC), isopropanol (IPA), potassium hexacyanoferrate(III) ACS reagent (≥99.0%), and potassium hexacyanoferrate(II) trihydrate (≥99.95%) were purchased from Sigma-Aldrich Canada Co. (Oakville, ON, Canada). Phosphate-buffered saline PBS (10x), pH 7.4 and 1x TRIS-buffered saline (TBS, 10x) pH 7.4 were purchased from Thermo Fisher Scientific, Mississauga, ON, Canada. Tween 20 was purchased from Alfa Aesar (Haverhill, MA, USA). Monoclonal Mouse IgG1 Clone SARS-CoV-2 Spike RBD antibody was purchased from Cedarlane lab, Burlington, NC, USA. The SYLGARD 184 silicone elastomer kit was purchased from DOW, Washington, DC, USA. Commercially available fat free skim milk was used without further purification. Ultrapure water (18.2 MΩ · cm at 25 °C) was obtained from Millipore equipment (Milli-Q water) for sample preparation and washing.

### 2.2. SARS-CoV-2 Spike Protein and Serum Samples

SARS-CoV-2 Spike protein was provided by Canadian Food Inspection Agency, in Dr. Shawn Babiuk’s lab, Winnipeg, MB R3E 3M4, Canada. The preparation, purification, and functional evaluation of SARS-CoV-2 spike protein has been described in detail in an earlier publication [43,72,73]. The anti-SARS-CoV-2 antibody positive and negative serum samples were obtained from the lab of Drs. Carmen Charlton and Jamil Kanji, Alberta Precision Laboratories, Calgary, AB T2N 1M7, Canada. Blood samples of patients used for the preparation of serum samples have been tested and confirmed to be either COVID-19 positive or negative by reverse transcription–polymerase chain reaction (RT–PCR). In addition, the serum samples have been tested and confirmed for the presence or absence of COVID-19 antibody by Alberta Precision Laboratories (APL) using two different assays, Abbott ARCHITECT SARS-CoV-2 IgG (Nucleocapsid) and DiaSorin LIAISON SARS-CoV-2 IgG (Spike).

### 2.3. Instruments, Electrical Contact Pad, and PDMS Mask

The potentiostat/galvanostat SP-200 BioLogic and associated EC lab software was from Science Instruments Inc. (Knoxville, TN, USA). Sirus T2 Tabletop Reactive Ion Etcher was from Trion Technology, Clearwater, FL, USA was used plasma cleaning of the chips. Custom-built electrical contact pad with a connector adapter used for the Electrochemical Impedance Spectroscopy (EIS) measurements of the Au-IDA chip sensors has been described earlier [43,72,73].

### 2.4. IDA Fabrication and Functionalization

The procedure adapted for the fabrication of gold IDA Chip on borofloat glass wafers using standard lift-off microfabrication technique has been described earlier [43,72,73]. Each Au-IDA chip has eight IDAs per chip which consists of 500 interdigitated comb electrodes arranged in an area of 3 mm × 3 mm. The interdigitated comb electrodes in the Au-IDA have a thickness, width, and gap of 70 nm, 4 µm, and 2 µm, respectively. Cleaning of the Au-IDA chips was achieved by sonicating in AC, IPA, DI water for 5 min and drying in N_2_ flow. Then, the chips were exposed to a Reactive Ion Etching (RIE) instrument for plasma treatment (100 sccm O_2_, 100 mT pressure, 98 mT oxygen, 100 W RF, 120 s) to burn off trace organics impurities on the surface. The cleaned Au-IDA chips were incubated in 1 mM MHA in EtOH solution at room temperature overnight to form a densely packed MHA monolayer and washed with EtOH followed by DI water and drying with N_2_.

### 2.5. Faradaic EIS Drift Signal Stabilization Protocol

The freshly prepared MHA monolayer coated Au-IDA chips were treated with a protocol we developed in this report to suppress the measurement induced change in EIS spectrum as determined by the R_ct_ value. The implementation of the protocol is accomplished in a single step and consists of incubation of the freshly prepared MHA-coated Au-IDA in 5 mM [Fe(CN)_6_]^−3/4^ 1x Tri-buffered solution at pH 7.4 and imposing bias voltage intermittently by scanning the EIS of it from 1Hz to 0.1 MHz by applying an AC sinusoidal voltage of ±10 mV superimposed on 0.4 V DC. Custom made PDMS mask was used to incubate/measure EIS of each Au-IDA on the chip separately. Application of the protocol led to an initial rapid decrease in R_ct_, calculated from the EIS spectrum, followed by relaxation to a stable value. The process typically requires 5 to 7 repeated scanning of EIS spectra in the span of ~2 h while incubating the MHA-coated Au-IDA in 5 mM [Fe(CN)_6_]^−3/4^ 1x Tri-buffered solution at pH 7.4. After the application of the protocol, the EIS spectrum as determined by the R_ct_ value remained stable with a coefficient of variation of <3% for several hours. The baseline drift-stabilized MHA-coated Au-IDAs were washed in DI water dried with N_2_ and stored in 1x PBST at pH 7.4 before use.

### 2.6. Spike Protein Immobilization

Immobilization of SARS-CoV-2 spike proteins on the baseline drift-stabilized MHA-coated Au-IDAs (MHA-Au-IDA) were carried out by using a PDMS mask which has eight matching square holes to form wells around the eight Au-IDAs on the chip and this allows MHA-Au-IDAs in the wells to be incubated with different solutions without intermixing [43,72,73]. The surface carboxylic acid group of the MHA-coated Au-IDA was activated by incubating for ~15 min in 50 µL aliquot of freshly prepared 73 mM EDC and 243 mM NHS solution in 10 mM MES buffer at pH 5.5. Consequently, after washing, it is incubated for 1 h in freshly prepared ~70 µg/mL SARS-CoV-2 spike protein in 10 mM PBST (1x PBS containing 0.05% Tween 20) at pH 7.4. After washing, the SARS-CoV-2 spike protein immobilized on baseline drift-stabilized MHA-coated Au-IDA (spike-MHA-Au-IDA) is used for analysis of anti-SARS-CoV-2 antibody samples.

### 2.7. Faradaic EIS Immunoassay of SARS-CoV-2 Antibodies

The PDMS mask with eight matching square holes which form wells around the eight Au-IDAs on the chip was used for incubation and measurement of EIS spectrum [43,72,73]. To measure the background signal, the spike-MHA-Au-IDA was incubated in 1% milk solution in 1x PBST at pH 7.4 at room temperature for ~1 h. The 1% milk solution is expected to prevent non-specific bindings on Spike-MHA-Au-IDA. After washing with 1x PBST at pH 7.4 solution, a 50 μL aliquot of 5 mM [Fe(CN)_6_]^−3/4^ 1x Tri-buffered solution at pH 7.4 was added and the EIS spectrum was measured by applying an AC sinusoidal voltage of ±10 mV superimposed on 0.2 V DC. The background signal ([R_ct_]_bgr_) of the analysis was determined from the Nyquist plot of the EIS spectrum. After washing, it is consequently incubated for ~1 h in the sample solution which is composed of a 50 μL aliquot of the desired dilutions of either IgG1 or serum solution in 0.1% milk solution in 1x PBST at pH 7.4. The EIS spectrum of the sample is measured in the same way as that of the background signal to obtain the corresponding sample signal, [R_ct_]_sample_. No systematic drift in the EIS spectrum was observed on repeated measurements of either the background or the sample in 5 mM [Fe(CN)6]^−3/4^ 1x Tri-buffered solution at pH 7.4. The EIS response of the sample or background, evaluated as R_ct_ from the Nyquist plot, to the presence or absence of IgG1 or anti-SARS-CoV-2 antibodies in the solution can be calculated from the relative response (RR) using the relationship RR = ([R_ct_]_sample_ − [R_ct_]_bgr_)/[R_ct_]_bgr_.

## 3. Results and Discussion

### 3.1. Interfacial Charge-Transfer and Dynamics in Freshly Prepared MHA Au-IDA

Chemical inertness of Au, biocompatibility, electronic conductivity, and ease of surface functionalization with thiols to form SAMs make Au an ideal transducer for electrochemical biosensors. The formation of SAMs with different thiols bound to Au and their properties have been extensively studied and characterized [74,75]. These studies have shown that even a highly oriented and densely packed SAM has two kinds of defects, pinholes and collapsed sites, due to imperfect adsorption of the thiols. Therefore, the SAM-coated electrode behaves like a microarray electrode [76,77,78,79,80,81,82,83,84,85,86,87]. Pinholes allow ions and solutes in the solution to have direct access to the electrode surface and the rate constant of heterogeneous electron transfer at these sites is similar to that of the bare Au electrode. In this system, the measured R_ct_ has contributions from a parallel combination of resistances from the pinholes, collapsed sites, and the full-length monolayer. According to Marcus theory [88], the electron transfer rate constant (k) decreases exponentially with thickness (d) of the insulating layer coating the electrode according to Equation (1).

*k* = *k*_0_*e^−βd^* (1)

where k_0_ is the heterogeneous electron transfer rate constants at the bare electrode, and β is the potential independent electron tunneling coefficient. The corresponding current (I) at the SAM-covered microarray electrode is given by Equation (2) [76,77,78,79,80,81,82,83,84,85,86,87].

*I* = *I*_0_*e^−βd^* (2)

where *I_o_* is the current measured at the bare electrode. Clearly, these equations indicate that current arising from the heterogeneous electron transfer at the pinholes of the microarray electrode dominates and the observed Rct can be described by Equation (3) [76,77,78,79,80,81,82,83,84,85,86,87].

(3) $$R_{ct}=\frac{RT}{{{n^{2}F}^{2}Ak}_{app}c\;}$$

where n is the number of electrons, R is the gas constant, T is the temperature, F is the Faraday constant, A is the total area of the electrode, and c is the concentration of the redox couple. The heterogeneous electron transfer rate constants of the bare (k_0_) is related to that of the modified electrode (k_app_) according to the relation (4),

k_app_ = k_0_(1 − θ) (4)

where θ is the fractional coverage of the electrode by the SAM.

In the EIS spectrum, the real (Z′) and the imaginary (Z″) part of the Nyquist plot can be fitted with Equations (5) and (6) [89,90]

(5) $$Z^{\prime }=R_{s}+\frac{R_{ct}}{1+\omega^{2}{R_{ct}^{2}C}_{s}^{2}}$$

(6) $$Z^{\prime \prime }=-\frac{\omega R_{ct}^{2}C_{s}}{1+\omega^{2}{R_{ct}^{2}C}_{s}^{2}}$$

where R_s_, R_ct_, and C_s_ are the solution resistance, charge transfer resistance, and sensor double layer capacitance, respectively. C_s_ can be obtained from the frequency (ω_max_) at which the magnitude of Z″ is maximum in the Nyquist plot and is given by Equation (7).

(7) $$C_{s}=\frac{1}{\omega_{max}R_{ct}}$$

The C_s_, in turn, is a parallel combination of capacitances from the fractions of SAM-modified (C_m_) and bare gold electrode (C_dl_) and is given by Equation (8).

(8) $$C_{s}=\theta C_{m}+(1-\theta )C_{dl}$$

**Figure 1:**
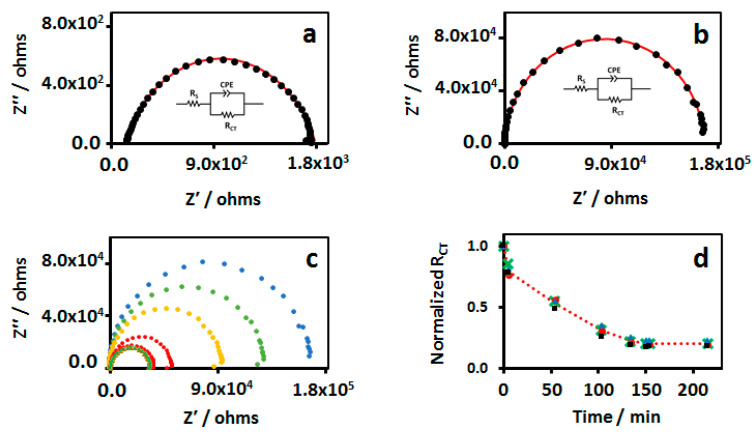
Dynamics of EIS for freshly prepared bare and MHA SAM-coated gold IDA. Initial EIS in Nyquist representations of (**a**) bare and (**b**) MHA SAM-coated gold IDA, and (**c**) EIS spectrum of MHA SAM-coated gold IDA successively recorded at different times. (**d**) Time dependence of normalized R_ct_ values due to the combined effect of incubation in 5 mM [Fe(CN)_6_]^−3/4^ containing 10 mM Tris buffer at pH 7.4 and intermittent EIS scanning for five different freshly prepared MHA SAM-coated gold IDAs. The red solid curve in (**a**,**b**) is the Randles equivalent circuit model (inset) fits to the experimental data.

Figure 1a shows the EIS spectrum of the plasma cleaned bare gold IDA recorded by excitation with an AC sinusoidal voltage of ±10 mV superimposed on 0.4 V DC and scanning the frequency from 1 Hz to 0.1 MHz in 5 mM [Fe(CN)_6_]^−3/4^ containing 10 mM Tris buffer at pH 7.4. The experimental data was simulated with a simplified Rundles equivalent circuit consisting of a parallel combination of a constant phase element (CPE) and R_ct_ in series with R_s_. The best fit represented by the red solid line was obtained with the following circuit parameters: (110 ± 10) ohms, 1655 ± 12 ohms, 0.78, and (1.0 ± 0.1) × 10^−6^ F · s^α−1^ for R_s_, R_ct_, α, and CPE, respectively. The C_dl_ obtained from the Nyquist (Equation (7)) plot was (6.8 ± 0.6) × 10^−6^ F · cm^2^, similar to that calculated from the CPE (C_dl_ = CPE · ω^α−1^) [91]. In the case of the clean bare IDA electrode, repeated scanning of the EIS was not observed to result in a gradual change in the R_ct_ value. Figure 1b shows the EIS spectrum of a freshly prepared MHA SAM-coated gold IDA. The SAM was prepared using gold IDA which was freshly cleaned by exposure to oxygen plasma by incubation in 1 mM MHA in EtOH at room temperature overnight. Figure 1b also shows the Nyquist plot and the best fit (red curve) with the Rundles equivalent circuit which yielded the following values for the circuit elements: 91 ohms, 168 kohms, 0.97, and 4.0 × 10^−8^ F · s^α−1^ for R_s_, R_ct_, α, and CPE, respectively.

A serious issue hampering the development of electrochemical biosensors based on SAM-functionalized gold electrodes with Faradaic detection using [Fe(CN)_6_]^−3/4^ as the redox reagent is the dramatic change in the signal on repeated measurements of the EIS spectrum. For a biosensor based on Faradaic detection, the change in the magnitude of the R_ct_ before and after binding of the target analytes determines the detection signal, and hence any drift in the R_ct_ value will adversely affect the magnitude of the detection signal and hence the reliability of the device. To suppress the drift in Rct of the sensor we have adopted a simple approach which consists of incubating the SAM-coated IDA in 5 mM [Fe(CN)_6_]^−3/4^ 1x Tri-buffered solution at pH 7.4 and imposing voltage intermittently by scanning the EIS of the system from 1Hz to 0.1 MHz while applying ACsinusoidal voltage of ±10 mV superimposed on 0.4 V DC. The effect of our protocol on the R_ct_ of the MHA SAM-modified IDA sensor is shown in Figure 1c. The Nyquist plots sequentially recorded at different times for a freshly prepared MHA SAM-coated gold IDA show an initial rapid decrease in R_ct_ followed by a slower decrease to a relatively stable value in about 2 h. The application of this protocol led to a sequential decrease in the R_ct_ value of the sensor from a high initial value of 168 kΩ to a stable value 34 kΩ. Figure 1d shows R_ct_(Norm) versus time plots of the experimentally determined response for five different MHA SAM-coated Au-IDAs on the application of the protocol presented above to stabilized the R_ct_ drift. The use of normalized R_ct_ value, where R_ct_(Norm) = R_ct_ (t)/R_ct_(t = 0), allows direct visual comparison of the dynamics of R_ct_ of different IDA electrodes. The R_ct_ of freshly prepared MHA-coated IDA electrodes decrease rapidly from the high initial values of 168, 158, 167, 155 and 174 kΩ to attain relatively stable values of 34, 32, 36, 36 and 32 kΩ, respectively, after application of the protocol. These results indicate that the proposed protocol is suitable for the suppression of the baseline signal drifts. The R_ct_ values of the stabilized sensors exhibited a low coefficient of variation (<3%), remaining stable during a few hours of incubation in a 5 mM [Fe(CN)6]^−3/4^ 1x Tris-buffered solution at pH 7.4. Furthermore, the stability persists even after incubation in a 1x Tris-buffered solution at pH 7.4 for several days. However, a prolonged incubation of the stabilized sensor in 5 mM [Fe(CN)_6_]^−3/4^ 1x Tri-buffered solution at pH 7.4 lasting several days was observed to lead to etching and dissolution of the gold electrodes. These results suggest that two mechanisms contribute to the change in the Rct value. The process contributing to the initial rapid change can be attributed to the reorganization of the SAM and the second process which operates concurrently is the slow etching of the gold electrode by CN^-^ ions produced by photoinduced dissociation of [Fe(CN)_6_]^−3/4^. The baseline drift-stabilized MHA SAM-coated Au-IDAs chips were stored at 4 °C in 10 mM PBST buffered solution at pH 7.4 and were used for the preparation of the sensor for the detection of COVID-19 antibodies.

### 3.2. Impedance Characterization of Defects in MHA Au-IDA

To gain further insight into the mechanism contributing to the processes occurring in the microelectrode array of SAM-coated Au-IDAs which is manifested as a dynamic change in the R_ct_ of the sensor, it is essential to experimentally determine the parameters controlling the interfacial electron transfer. Equations (3)–(8) provide the relationships among these parameters and to the real (Z′) and the imaginary (Z″) part of the Nyquist representation of the EIS spectrum of the sensor. The Faradaic impedances, Z_f_′ and Z_f_″, which accounts for the Faradaic contribution to Z′ and Z″, respectively, in the low-frequency case corresponding to nearly isolated diffusion profiles for each microelectrode in the Au-IDA are given by the following expressions [76,77,78,79,80,81,82,83,84,85,86,87]:

(9) $$Z_{f}^{\prime }\left(\omega \right)=\frac{R_{CT}}{1-\theta \;}+\frac{\sigma }{\sqrt{\omega }}+\frac{\sigma r_{a}{\left(0.72D\right)}^{1/2}}{\left(1-\theta \right)}$$

(10) $$Z_{f}^{\prime \prime }(\omega )=\frac{\sigma }{\sqrt{\omega }}$$

where σ is Warburg coefficient, r_a_ is the radius of the pinhole in the SAM-coated IDA, and D is the diffusion coefficient (7.3 × 10^−6^ cm^2^/s) of the redox couple [Fe(CN)_6_]^−3/4^.

**Figure 2:**
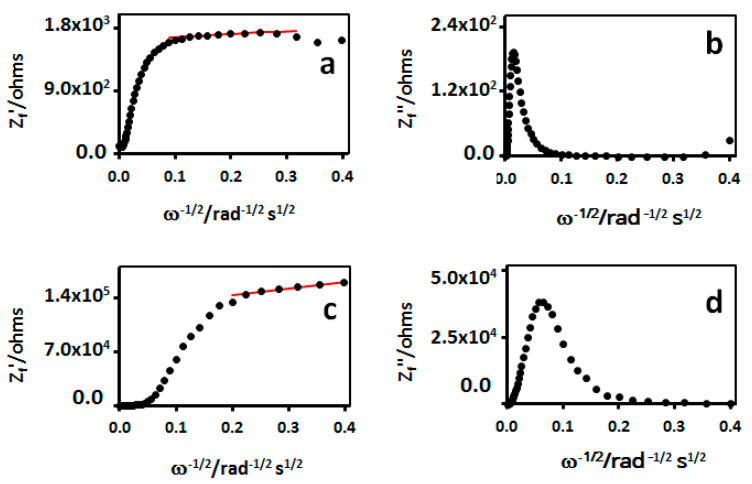
Dependence of Z_f_′ and Z_f_″ on frequency for bare gold IDA and for freshly prepared MHA SAM-coated IDA. Plots of (**a**) Z′_f_ versus ω^−1/2^ and (**b**) Z_f_″ versus ω^−1/2^ for bare gold IDA. Plots of (**c**) Z_f_′ versus ω^−1/2^ and (**d**) Z_f_″ versus ω^−1/2^ for MHA SAM-coated IDA. The solid red lines in (**a**,**c**) correspond to the linear fit to the experimental data. The EIS spectra of the sensors were recorded in 5 mM [Fe(CN)_6_]^−3/4^ 1x Tri-buffered solution at pH 7.4.

Figure 2a shows the Z_f_′ versus ω^−1/2^ plot of the bare gold IDA sensor recorded by excitation with an AC sinusoidal voltage of ±10 mV superimposed on 0.4 V DC in 5 mM [Fe(CN)_6_]^−3/4^ 1x Tri-buffered solution at pH 7.4. The linear fit to plot yields a slope of 335.5 Ωs^−1/2^cm^2^ that corresponds to the Warburg coefficient (σ_Au_) of the bare gold IDA sensor, and corresponding value (σ_SAM_) for the MHA-coated gold IDA sensor can be obtained from the Z_f_′ versus ω^−1/2^ plot in Figure 2c.

**Scheme 1:**
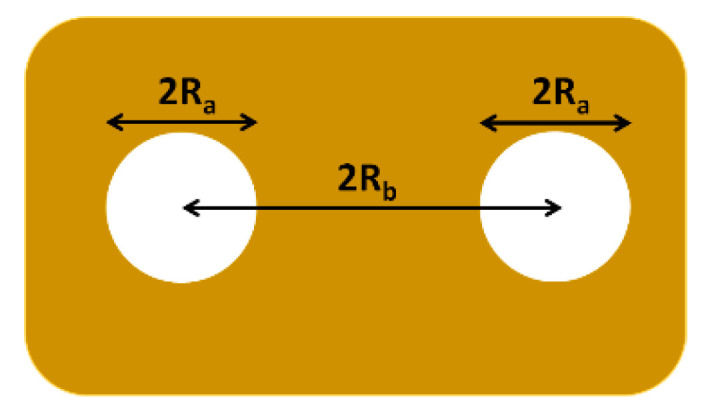
Illustration of pinholes and their separation in MHA Au-IDA. R_a_ and R_b_ are the radius of pinhole and their center-to-center separation distance, respectively.

The Warburg coefficients evaluated from the Z_f_′ vs. ω^−1/2^ plots for the bare gold electrode (σ_Au_) and the MHA-coated gold IDA electrode (σ_SAM_) can be used to calculate the fractional coverage (θ) of the monolayer when the SAM coverage is high (θ > 0.9) according to the relationship (11) [86]

(11) $$\theta =1-\frac{\sigma_{Au}}{\sigma_{SAM}-\sigma_{Au}}$$

which in turn is related to pinhole radius (R_a_) and half the distance between the centers of the adjacent pinholes or pinhole separation radius (R_b_) in the monolayer coated gold IDA electrode, as depicted in Scheme 1, and described by the relationship given below, Equation (12).

(12) $$1-\theta =\frac{R_{a}^{2}}{R_{b}^{2}}$$

and the radius of the pinhole (R_a_) can be determined from the frequency (ω_max_) at which Z_f_″ vs. ω^−1/2^ plot is maximum according to the expression (13):

(13) $$\omega_{max}=\frac{1}{2}\frac{D}{{0.36R}_{a}^{2}}$$

The parameters controlling the dynamics of R_ct_ due to the presence of pinholes and collapsed sites in the MHA-coated SAM on Au-IDA experimentally determined from the EIS characterization of the sensor are listed in Table 1. The figure of merit for sensors based on Faradaic detection is the R_ct_. It exhibits significant instability in the case of freshly prepared MHA-modified Au-IDA, as indicated by a >4x decrease in Rct value during repeated measurements of the EIS spectrum. Consequently, a freshly prepared MHA-modified Au-IDA is not suitable for Faradaic detection-based sensor applications, as it would result in substantial baseline signal drift. Upon application of the proposed protocol, the R_ct_ rapidly decreases to a relatively stable value in about 2 h where it remains stable for several hours in 5 mM [Fe(CN)_6_]^−3/4^ 1x Tri-buffered solution at pH 7.4. During this period, the sensor double-layer capacitance (Cs) is unaffected and the surface fractional coverage (**θ**) exhibits a small decrease. On the other hand, the principal contributor to the R_ct_ which is characterized by the defects in the MHA modified Au-IDA, the pinhole (R_a_) and pinhole separation radius (R_b_), exhibit remarkable initial rapid decrease to a relatively stable value during this period.

**Figure 3:**
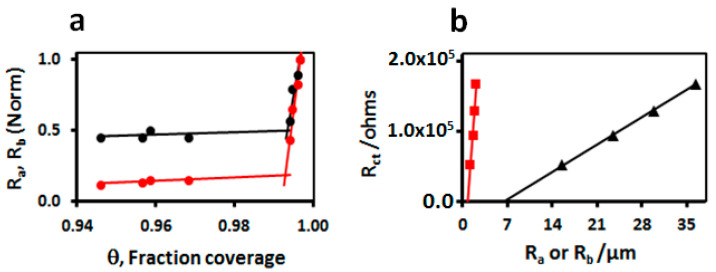
Correlations of charge-transfer resistance with defects and surface coverage in MHA modified IDA. (**a**) (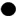) R_a_ versus θ and (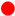) R_b_ versus θ plots. (**b**) (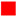) R_ct_ versus R_a_ and (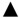) R_ct_ versus R_b_ plots. The solid lines are linear regression of the experimental data.

Figure 3 shows the plots of R_a_ versus θ, R_b_ versus θ, R_ct_ versus R_a_, and R_ct_ versus R_b_ to further explore the correlations between these parameters. The empirically observed correlations between θ and R_a_ and R_b_ are R_a_ = 0.83θ − 0.32, R_b_ = 1.61θ − 1.41 for the lower-coverage region 0.94 < θ < 0.96; and R_a_ = 159.74θ − 158.18 and R_b_ = 215.40θ − 213.67 for the high-coverage region θ > 0.99. These results show both R_a_ and R_b_ decrease rapidly with θ when the coverage is high θ > 0.99 but decrease slowly when the coverage is lower θ < 0.96. Figure 3b shows the dependence of R_ct_ on R_a_ and R_b_ in the high-coverage region θ > 0.99. Linear regression of the data yields the correlations, R_ct_ = 1.23 × 10^5^ R_a_ − 1.02 × 10^5^ and R_ct_ = 5.6 × 10^3^ R_b_ − 3.68 × 10^4^. Interestingly, the R_ct_ is ~22 times more sensitive to R_a_ than R_b_ and both R_a_ than R_b_ decrease with a decrease in θ. Thus, a decrease in surface coverage of MHA monolayer on gold IDA led to a surface which have a higher density pinhole with smaller radius. These results clearly demonstrated that the evolution of the R_ct_ in the early time scale is dominated by contributions arising from reorganization of the defect sites without loss of thiol SAM through desorption, oxidation, and gold dissolution. Reorganization of the collapsed sites is promoted by the applied bias field and the highly polar dielectric aqueous medium which combined to induce torque to the thiolate head groups and concurrent hydrophobic interactions facilitate the formation of homogeneous crystalline film in the SAM. Thus, from the mechanistic perspective, the applied bias voltage induces a torque to the thiolate head group driving a reorganization process in the SAM layer, facilitating crystallization of the SAM layer due to hydrophobic interactions, leading to a decrease in the number of collapsed sites concurrent with the generation of new pinholes which were presumably previously covered by the collapsed sites, and induces a decrease in the existing pinhole radius, without loss of the thiols, consequently resulting in a relatively small change in the fractional coverage. These results also indicate that CN^−^ induced etching of the Au surface leading to a loss of the SAM layer does not contribute to the observed R_ct_ change in the short time scale and becomes important in the much longer time scale.

### 3.3. Standard Curve for Anti-SARS-CoV-2 Antibody Test Using MHA Au-IDA Biosensor

**Scheme 2:**
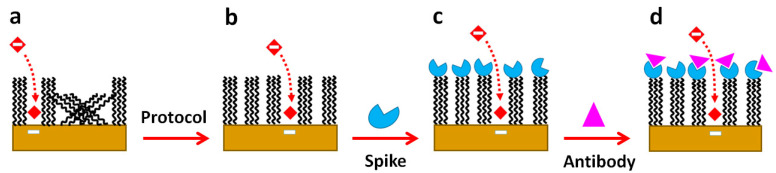
Cross sectional view of the sensor surface to illustrate the main steps in building a Faradaic immunosensor. (**a**) Freshly prepared MHA Au-IDA with well-ordered crystalline, pinhole, and collapsed site defects, (**b**) pinholes and well-ordered crystalline SAM after the collapsed site reorganization and crystallization, (**c**) after spike protein immobilization and incubation in with milk, and (**d**) after incubation in a serum sample containing anit-SARS-CoV-2 antibody. (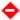) [Fe(CN)_6_]^−4^, (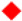) [Fe(CN)_6_]^−3^, (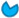) spike, and (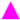) anit-SARS-CoV-2 antibody. Dashed arrow shows diffusion of [Fe(CN)_6_]^−4^ in the pinhole to approach the bare gold surface for interfacial electron transfer.

Sensors for COVID-19 antibody tests were prepared from the baseline drift-stabilized MHA SAM-modified Au-IDAs using the protocol presented in the previous section. Scheme 2 presents the main steps employed in this work to suppress the baseline signal drift and reliably detect the target anti-SARS-CoV-2 antibodies in human serum samples using the immunosensor with Faradaic detection. As depicted in the scheme, steric hindrance caused by the binding of the target analytes to the recognition elements on the transducer impedes diffusion of the redox probes onto the surface of the bare electrode through the pinholes, thereby affecting the impedance response which serves as the detection signal.

**Figure 4:**
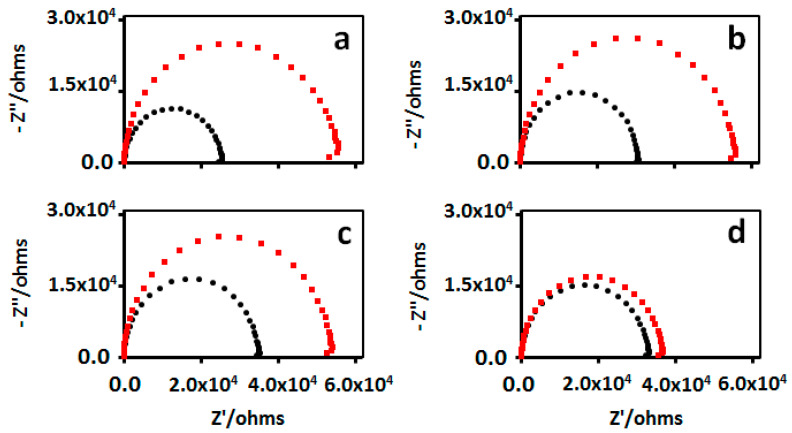
Nyquist plots for different concentrations of IgG1. EIS spectra were recorded after incubation for 1 h in (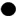) 1% milk solution containinng 1x PBST at pH 7.4 and then in (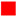) different concentrations of IgG1 in 0.1% milk solution containing 1x PBST at pH 7.4. IgG1 concentrations were (**a**) 50 µg/mL, (**b**) 5 µg/mL, (**c**) 0.5 µg/mL, and (**d**) 0.05 µg/mL. EIS spectra were recorded in in 5 mM [Fe(CN)_6_]^−3/4^ 1x Tri-buffered solution at pH 7.4.

The free terminal functional carboxylic acid groups in the MHA SAM-modified IDAs were activated with EDC/NHS chemistry to covalently conjugate with the surface amine groups on the spike proteins of SARS-CoV2 which serve as the recognition element of the sensor. It may be insightful to note that the spike glycoprotein is a homotrimer with each ~180-kDa monomer composed of S1 and S2 subunits consisting of 1273 amino acid residues anchored on the viral membrane [92]. A recent study showed that it has 145 epitopes but due steric and protective glycan shielding only about 10 epitopes which are exposed on the surface of the spike protein may be accessible to bind to the antibodies and suitable for use as neutralizing antibodies and therapy development [93,94,95,96]. The SARS-CoV-2 Spike RBD (Monoclonal Mouse IgG1) antibody used in this test binds to the SARS-CoV-2 Spike RBD located in the C-terminal domain of the S1 subunit which contains two domains, called the N-terminal domain (NTD) and C-terminal domain (CTD). In this test, each dilution of the monoclonal mouse IgG1 titer was analyzed in a separate freshly prepared IDA. As illustrated in Figure 1d of the previous section, the application of the protocol yields IDAs with different baseline drift-stabilized R_ct_ values. A quantitative detection of different dilutions of IgG1 in the prepared sample solutions was achieved in two steps, incubation of the IDA in 1% milk containing 1x PBST at pH 7.4 at room temperature (to block non-specific binding) and measuring the EIS response ([R_ct_]_bgr_) in 5 mM [Fe(CN)_6_]^−3/4^ 1x Tri-buffered solution at pH 7.4 followed by incubation of the IDA in the sample solution containing the desired IgG1 concentration in solution containing 0.1% milk in 1x PBST at pH 7.4 at room temperature and measuring the EIS response ([Rct]_sample_) in 5 mM [Fe(CN)_6_]^−3/4^ 1x Tri-buffered solution at pH 7.4. The calculated relative response, RR = ([Rct]_sample_ − [Rct]_bgr_)/[Rct]_bgr_, was used to quantify the IgG1 concentration in the sample solution. Figure 4 shows the Nyquist plots for four different concentrations of IgG1 obtained using different IDAs.

**Figure 5:**
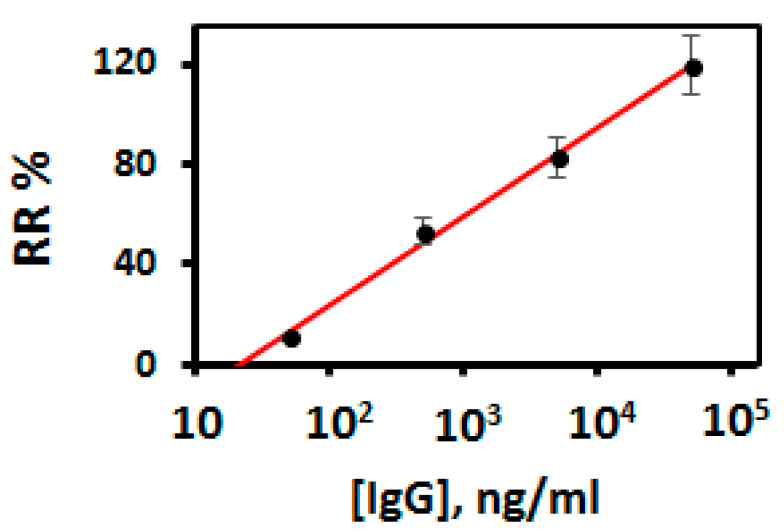
Plot of RR% versus IgG concentration. Impedance response to different concentrations of IgG1 in sample solutions containing 0.1% milk and 1x PBST at pH 7.4. (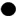) Experimental data, (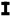)% relative standard deviation (*n* = 3), and linear regression (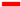) to the experimental data points yielded RR% = 35.43 log [IgG] − 46.98, R^2^ = 0.995.

Figure 5 shows the plot of RR% versus IgG1 concentration in the sample solutions containing 0.1% milk and 1x PBST at pH 7.4. The RR% data points were calculated from the experimentally determined R_ct_ values obtained from the Nyquist plots as shown in Figure 4 using the relation, RR = ([R_ct_]_sample_ − [R_ct_]_bgr_)/[R_ct_]_bgr_, where [R_ct_]_sample_ and [R_ct_]_bgr_ are the charge-transfer resistances obtained for the sample and background solutions, respectively. Linear regression of the experimental data points yields the assay sensitivity of 35.4/decade (35.4%/10 ng mL^−1^) and limit-of-detection (LOD) of 21 ng/mL evaluated from extrapolation of the linear fit to the 3.3x signal-to-noise ratio of the background. A performance parameter of huge importance to electrochemical biosensors is the relative sensitivity of Faradaic and non-Faradaic detection. From the magnitudes of the slopes of the standard curves of the Faradaic and non-Faradaic biosensor, the sensitivity of the Faradaic biosensor is estimated to be ~17 times that of the non-Faradaic biosensor.

Table 2 lists the LODs of the recently developed electrochemical biosensors based on Faradaic detection using [Fe(CN)_6_]^−3/4^ as the redox reagent for detection and quantification of anti-SARS-CoV-2 IgG antibody. (Liv et al.) reported the lowest LOD of 9.3 ag/mL and other remarkably low LODs are 1fg/mL and 0.4 pg/mL, which were reported by Sadique et al. [48] and Cardoso et al. [51], respectively. However, it is interesting to note that most of the LODs reported as listed in Table 2 for anti-SARS-CoV-2 IgG antibody detection are in the ng/mL range (1 nM corresponds to 150 ng/mL for IgG). It worth noting that attaining a low LOD for electrochemical biosensors to a range relevant to the concentration of biomarker in the bio-fluid of the patient is one of the major constraints for their commercialization. Thus, these remarkably low LODs for anti-SARS-CoV-2 IgG antibody quantification, if validated, will certainly raise the prospects of commercializing electrochemical biosensors.

The molecular weight of IgG, 150 kDa, was used to convert concentration from M to g/mL. Equation relating WHO IS antibody concentration to nanogram/mL (ng/mL) is given by BAU/mL = ng/mL × Conversion Factor, and the Conversion Factor values are 0.0027, 0.0022, and 0.0142 for RBD specific to IgG, IgG specific to spike S1, and RBD specific to IgM, respectively [97].

### 3.4. Performance of MHA Au-IDA Biosensor to Detect Anti-SARS-CoV-2 Antibodies in Human Serum

**Figure 6:**
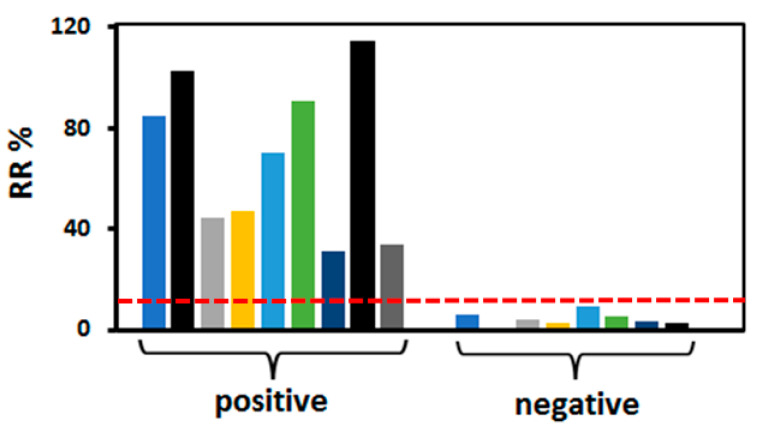
Tests of clinical human serum samples from nine SAR-CoV-2 antibody positive and eight SAR-CoV-2 antibody negative patients using MHA Au-IDA biosensor. Serum samples of the patients have been tested and confirmed for the presence or absence of SARS-CoV-2 virus and anti-SARS-CoV-2 antibodies using nucleic acid and serology tests, respectively. Dashed line indicates the LOD.

Impedance response to different concentrations of anti-SARS-CoV-2 IgG1 antibody as presented by the standard calibration curve (Figure 5) indicates the potential suitability of the developed biosensor for determining the presence/absence of anti-SARS-CoV-2 antibodies in human serum samples. Figure 6 shows the test results obtained using MHA Au-IDA biosensor for serum samples collected from nine patients which have COVID-19 antibody and eight patients which do not have COVID-19 antibody as tested using a commercial serology assay. These patients have also been confirmed to be either COVID-19-positive or COVID-19-negative using a nucleic acid test [98,99,100,101]. A protocol similar to that used for the determination of the standard curve is adopted to test clinical human serum samples. Figure 6 shows that the impedance response (RR%) varies with patient, indicating the varying amount of antibodies present in the sera as expected. For patients which have tested anti-SARS-CoV-2 antibody positive, the RR% values are significantly above the dotted line indicating the LOD of the biosensor and, on the other hand, for patients which have tested anti-SARS-CoV-2 antibody negative, the RR% values are below the LOD as expected. Based on the limited number serum samples analyzed in this study, the sensitivity and specificity of the biosensor can be estimated to be 100%.

The observed high sensitivity and specificity of the biosensor suggest negligible or no cross reactivity of the biorecognition element with non-target antibodies or other biomolecules present in the serum. These parameters depend on the intrinsic properties of the SARS-CoV-2 spike protein and a literature review indicates that biosensors using the spike protein as the biorecognition element also report no cross reactivity with the seasonal human coronavirus such as HCoV-229E, HCoV-OC43, HCoV-NL63 and HCoV-HKU1 and other endogenous antibodies present in serum. Another important characteristic of a biosensor for POC applications is the analysis turnaround time which is predominantly determined by the incubation time. Incubation periods typically require several hours compared to <1 min which is required to record an EIS spectrum as displayed in Figure 1 and Figure 4. To determine the suitability of the MHA Au-IDA biosensor for POC applications we tested an incubation time of 10 min and determined that the biosensor is still capable of detecting the antibodies in human serum with no significant effect on the impedance response as determined by the RR% value.

## 4. Conclusions

In this work, we have highlighted the problem associated with Faradaic detection using [Fe(CN)_6_]^−3/4^ as the redox reagent in an electrochemical biosensor based on SAM-modified Au electrodes and presented a protocol to resolve the challenge. We have demonstrated that the dramatic change in Faradaic current observed in freshly prepared SAM-modified Au electrodes arises from the bias-induced reorganization of SAM and this change can be inadvertently attributed to the detection signal associated with the presence of target analytes in the sample. We have also provided insights into the mechanisms responsible for the dynamic reorganization of SAM on Au electrodes. Based on experimental data on the evolution of the pinhole radius and density in the system, we determined that the pinhole radius and separation decrease as the SAM fraction coverage of the electrode decreases. The results also showed that CN^−^ ion etching of Au occurs at a relatively slower rate compared to the reorganization of SAM under the application of the proposed protocol, thereby providing the biosensor with a window of time for baseline drift-free analysis. By applying the protocol, we have demonstrated a baseline signal drift-stabilized label-free MHA Au-IDA biosensor with Faradaic EIS detection for analysis and quantification of anti-SARS-CoV-2 antibodies in human serum samples. The label-free biosensor developed in this work requires a short incubation time of 10 min, provides a sensor sensitivity of 35.4/decade and LOD of 21 ng/mL. In addition to high sensitivity, the biosensor was also shown to be highly specific with negligible cross reactivity to seasonal human coronavirus or endogenous antibodies. Remarkably, a comparison of the detection sensitivity showed that Faradaic biosensors are about 17 times more sensitive than non-Faradaic biosensors, indicating the potential of such systems for POC applications.

## Figures and Tables

**Table 1:** EIS characterization of the evolution of defects in MHA-coated SAM on Au IDA. Charge-transfer resistance (R_ct_), fractional coverage (θ), sensor double-layer capacitance (C_s_), pinhole radius (R_a_), and pinhole separation radius (R_b_). The values have relative standard deviation (RSD) of ca. 10%. The Warburg coefficient (σ_Au_) of the bare gold IDA sensor is 335.5 Ωs^−1/2^cm^2^.

Time/Min	R_ct_/kohm	θ	C_s_, µF/cm^2^	R_a_, µm	R_b_, µm
0	168.0	0.996	1.34	2.14	36.33
5	129.6	0.995	1.38	1.90	29.95
54	94.0	0.994	1.51	1.70	23.48
103	51.4	0.992	1.38	1.20	15.58
134	37.2	0.959	1.53	1.07	5.26
150	33.9	0.965	1.33	0.95	4.10
154	34.5	0.957	1.30	0.95	4.58
214	34.1	0.968	1.32	0.95	5.36

**Table 2:** Electrochemical biosensors using [Fe(CN)_6_]^−3/4^ redox reagent for detection of SARS-CoV-2 antibodies and their analytical parameters. Gold nanocrystal (AuNC), glassy carbon electrode (GCE), graphene oxide (GO), screen-printed electrode (SPE), Fluorine-doped tin oxide–zinc oxide nanorod (FTO-ZnONR), single-walled carbon nanotube (SWCNT), gold nanoparticle (AuNP), 6-mercapto-1-hexanol (MHOH), cysteamine (CysNH2), electrochemical paper-based analytical device (ePAD). Cyclic voltammetry (CV), differential pulse voltammetry (DVP), pulsed potential amperometry (PPA), electrochemical impedance spectroscopy (EIS), square-wave voltammetry (SWV).

Electrode	Electrode Modification	Technique	Recognition Element	Analyte	LOD	References
Au-AuNC	MUA-MHOH/EDC/NHS	CV	Spike	IgG	51.0 ng/mL	Zukauskas et al. [44]
		DPV	Spike	IgG	91.5 ng/mL	
		PPA	Spike	IgG	105.0 ng/mL	
Au	MUA-MHOH/EDC/NHS	CV	Spike	IgG	379.5 ng/mL	Liustrovaite et al. [45]
		EIS	Spike	IgG	298.5 ng/mL	
GCE/Au	CysNH2/EDC/NHS	SWV	Spike	IgG	9.3 ag/mL	Liv et al. [47]
GCE-GO−Au	GO-COOH/EDC/NHS	DPV	N-protein	IgG	1.0 fg/mL	Sadique et al. [48]
Au/rGO	rGO-COOH/EDC/NHS	EIS	S1 spike	IgG	0.42 pg/mL	Ali et al. [49]
		EIS	RBD	IgG	2.54 pg/mL	
ePAD-GO	GO-COOH/EDC/NHS	SWV	RBD	IgG	0.96 ng/mL	Yakoh et al. [50]
SPE-SWCNT	CNT-COOH/EDC/NHS	EIS	Spike	IgG	0.7 pg/mL	Cardoso et al. [51]
FTO-ZnONR	Physisorbed/Electrostatic	EIS	Spike	IgG	19.34 ng/mL	Nunez et al. [52]
GCE-AuNP	Physisorbed	EIS/SWV	Spike	IgG	0.2 μg/mL	Castro et al. [53]
Au IDA	MHA/EDC/NHS	EIS	Spike	IgG	21 ng/mL	This work
Au IDA	MUA/EDC/NHS	Non-Faradaic	Spike	IgG	148.1 ng/mL	Shoute et al. [43]

## Data Availability

The data files including raw data used to support the findings in this study can be obtained from the corresponding author upon reasonable request.
